# A Case of Violent Suicide Attempt in a Context of Myxedema Psychosis following Radioiodine Treatment in a Patient with Graves' Disease

**DOI:** 10.1155/2019/4972760

**Published:** 2019-01-09

**Authors:** Louise Todorov, Amel Ait Boudaoud, Rachel Pascal de Raykeer, Alina Radu, Khadija Lahlou-Laforêt, Frédéric Limosin, Cédric Lemogne, Sébastien Czernichow

**Affiliations:** ^1^AP-HP, Hôpitaux Universitaires Paris Ouest, Service de Psychiatrie de l'Adulte et du Sujet Âgé, Paris, France; ^2^AP-HP, Hôpitaux Universitaires Paris Ouest, Service de Nutrition, UF Endocrinologie-Diabétologie, Paris, France; ^3^Université Paris Descartes, Sorbonne Paris Cité, Faculté de Médecine, Paris, France; ^4^Inserm, U894, Centre Psychiatrie et Neurosciences, Paris, France

## Abstract

**Introduction:**

Hypothyroidism has been associated with mood disorders but some cases of acute psychosis have also been reported. However, less attention has been paid to suicidal behavior in these patients.

**Case Report:**

We report a case of suicide attempt by self-stabbing in a 43-year-old woman without past psychiatric history, four months after radioiodine therapy for Graves' disease. On clinical examination remarkable signs of myxedema were found and blood investigations showed hypothyroidism with an extremely high thyroid stimulating hormone (TSH) level (152 mUI/L; reference range 0.20-5.10). The patient presented delirium symptoms at the time of self-stabbing, which was associated with persecutory delusions and auditory harm command hallucinations. A rapid physical and psychiatric improvement was observed after the initiation of an oral thyroid replacement therapy without relapse after early discontinuation of the antipsychotic treatment.

**Discussion:**

The most distinctive feature of our case is that the violent suicide attempt could be attributed to the myxedema psychosis. Suicide may result from several factors, including psychosocial stressors, psychiatric symptoms, and hormonal disturbance. This unique presentation should remind clinicians to systematically consider ordering additional tests in patients with atypical psychiatric presentation, even when serious behavioral disorders (such as violent suicide attempts) are present and may result in premature transfer to psychiatric units.

## 1. Introduction

Hypothyroidism has commonly been associated with mood disorders, particularly depression, but a few cases of acute psychosis have also been reported. Since the description of myxedema psychosis by Asher in 1949 [1], various psychotic symptoms such as delusions and hallucinations have been observed. To our knowledge, suicidal behaviors have never been described in these patients. We report herein a unique case of violent suicide attempt by self-stabbing in a patient with myxedema psychosis.

## 2. Case Report

A 43-year-old woman without psychiatric or substance use disorder history was admitted in a thoracic surgery unit for a suicide attempt by self-stabbing. She had been treated for Grave's disease by carbimazole for six years. Almost four months before the suicide attempt, she received radioiodine therapy. Because of a misunderstanding, carbimazole was not switched by the patient for levothyroxine but had been continued until one week before admission. She presented three self-inflicted knife wounds, causing a hemopneumothorax and a pulmonary contusion. The initial clinical evaluation reported a significant exophthalmos, a cutaneous myxedema, and a hoarse voice. She had a normal-size thyroid gland without nodule. A consultation-liaison psychiatrist's evaluation was requested on day one, before chest drainage under general anesthesia.

The first evaluations showed that the patient was disoriented with incoherent speech and intermittent agitation, compatible with mild delirium. She did not express suicidal ideation and did not remember stabbing herself. However, she clearly reported harm command hallucinations. Her relatives described behavioral changes seven days before the admission, including sleep disorders and persecutory delusions; she thought that she was under surveillance by her employer. Just before the suicide attempt, which occurred at night, she tried to leave the house naked.

Blood investigations on day three showed an extremely high thyroid stimulating hormone (TSH) level (152 mUI/L; reference range 0.20-5.10), low free thyroxine (fT4) level (1.5 pmol/L; reference range 11-24), and low free tri-iodothyronine (fT3) level (< 1.3 pmol/L; reference range 2.5-7). Thyroid peroxidase (1712 UI/ml; reference range <40), thyroglobulin (192 UI/ml; reference range <120), and anti-TSH receptor antibodies (6 UI/L; reference range < 1.6) levels were also elevated. The electroencephalogram and the cerebrospinal fluid on day six were normal. A brain magnetic resonance imaging scan showed nonspecific subcortical hyperintensities in the white matter. A thyroid ultrasound revealed signs of thyroiditis, without nodule.

Oral antipsychotic treatment (chlorpromazine 100 mg per day) was initiated on day three to reduce both the impulsivity and anxiety. On day seven, the patient also started oral thyroid replacement therapy (levothyroxine 50 *μ*g per day for seven days, then 100 *μ*g per day for two days, and finally 125 *μ*g per day) because of a persistent increase of the TSH level. The psychotic symptoms rapidly disappeared between day 14 and day 19 and did not uncover any underlying mood disturbance. The cutaneous myxedema drastically reduced on day 18. The antipsychotic treatment was gradually stopped from day 19 to day 25. Endocrine blood tests on day 22 showed TSH level at 83.1 mUI/L and fT4 level at 8.4 pmol/L. The patient was discharged on day 32.

At follow-up visits one week and one month after discharge, the patient was asymptomatic and the TSH level was 22mUI/L and 8.37mUI/L, respectively. She did not remember in detail the suicide attempt but could express a feeling of “not being herself” at that time.

## 3. Discussion

Here we report a unique case of violent suicide attempt in a context of acute myxedema psychosis. Myxedema psychosis was diagnosed based on several elements: no past psychiatric history, emergence of psychiatric symptoms after radioiodine therapy, severe hypothyroidism, remarkable signs of myxedema, rapid improvement after the initiation of a thyroid replacement therapy, and absence of relapse after the early discontinuation of the antipsychotic treatment. Furthermore, Hashimoto's encephalopathy, which would have warranted a treatment with corticosteroids, was ruled out based on the clinical picture and the normal electroencephalogram. Regarding the duration between radioiodine therapy and psychosis (three to four months), the rapid improvement after the initiation of a thyroid replacement therapy (within one to two weeks), and the absence of relapse at follow-up, similar cases have been described [2–4]. Compared to these cases, the most striking feature of our patient was the violent suicide attempt by self-stabbing, which may be attributed to the myxedema psychosis because of the lack of any past psychiatric episode or other psychiatric diagnosis. This event occurred in a context of multiple psychosocial stressors, including a conflict with her employer and persistent domestic violence, for which she was in the process of getting a divorce. According to her relatives, however, she had never expressed suicidal ideations. Moreover, the patient did not remember the suicidal act but reported harm command hallucinations before the suicide attempt. The patient's relatives also reported that the patient had persecutory delusions one week before the suicide attempt. The amnesia, altered awareness, and intermittent agitation of the patient when she was admitted may also suggest delirium symptoms at the time of stabbing.

This violent suicide attempt might therefore result from the interplay of different factors, including chronic psychosocial stressors, but also and above all persecutory delusions, harm command hallucinations, and delirium leading to behavioral disinhibition and increased impulsivity. A direct role of the hormonal disturbances in the suicidal behavior of this patient may also be discussed. Lower fT3 values have been associated with a recent history of suicide attempts regardless of psychiatric diagnosis in some clinical studies (e.g., [5]). In depressed patients, lower basal fT4 values [6] or deficits in central thyrotropin-releasing hormone (TRH) [7] have also been associated with a history of suicide attempt. Interestingly, a history of violent suicide attempts was specifically associated with a lowered TSH-TRH response [7]. Since the thyroid axis might be involved in the homeostasis of the serotonergic system, as suggested by animal models based on experimentally induced hypothyroid states [8], this lack of reactivity may contribute to suicidal behavior through abnormalities in the serotonin system [5, 9]. However, investigations on reduced TRH response have not yielded robust findings across clinical studies over the past decades. Further studies are needed to refine our understanding of the relationship between the thyroid axis and the serotoninergic system, as well as other biological systems potentially involved in suicidal risk (e.g., inflammation).

This case highlights a rare but serious consequence of severe hypothyroidism and allows us to identify major issues for the management of these patients. First, the endocrinologists and general practitioners, as well as the patients and their relatives, should be aware of early psychiatric symptoms that could reveal a worsening of a thyroid dysfunction, notably in case where thyroid replacement therapy has been interrupted. Here, the patient had seen her general practitioner twice the week before her admission. Likewise, the patients and their relatives should be informed of relevant neuropsychiatric complications of the thyroid hormonal disturbance. Second, mental health professionals should also be aware that psychiatric symptoms may be the first signs of severe hypothyroidism [10–12].

In this case of a violent suicide attempt of a patient facing psychosocial problems, consultation-liaison psychiatrists played a key role in preventing the behavioral disturbances being attributed solely to the psychological issues and in promoting the ordering of adequate additional tests.

Furthermore, it should be kept in mind that behavioral disturbances such as agitation or suicidal attempts, especially violent ones, can lead to a rapid transfer to a psychiatric ward for safety reasons prior to further investigations. In addition, the risk of underestimating an underlying physical condition might be increased when the patient presents suicidal thoughts. For instance, in the context of a psychiatric emergency department, suicidal ideation has been shown to moderate the likelihood of ordering biological tests associated with age [13]. In the literature, the patients were usually hospitalized in psychiatric wards before the diagnosis of hypothyroidism. This is likely to delay the initiation of an appropriate endocrine treatment and eventual clinical improvement. Therefore, thyroid function should be systematically assessed among patients with atypical psychiatric symptoms, and associated behavioral disorders, such as suicide attempts, should not precipitate premature transfer to a psychiatric unit.
